# Nitrogen and phosphorus addition differentially affect plant ecological stoichiometry in desert grassland

**DOI:** 10.1038/s41598-019-55275-8

**Published:** 2019-12-10

**Authors:** Lei Li, Bo Liu, Xiaopeng Gao, Xiangyi Li, Chengdao Li

**Affiliations:** 1 State Key Laboratory of Desert and Oasis Ecology, Xinjiang Institute of Ecology and Geography, Chinese Academy of Sciences, Urumqi, 830011 China; 2 Cele National Station of Observation and Research for Desert-Grassland Ecosystem in Xinjiang, Cele, 848300 Xinjiang China; 3 Shandong Provincial Key Lab. of Soil Conservation and Environmental Protection, College of Resources and Environment, Linyi University, Linyi, 276000 China; 4 Department of Soil Science, University of Manitoba, Winnipeg, Canada; 5 University of Chinese Academy of Sciences, Beijing, 100049 China

**Keywords:** Element cycles, Element cycles

## Abstract

Plant C:N:P stoichiometric relations drive powerful constraints on ecological interactions and processes. However, information about plant stoichiometric responses to N and P availability in desert grassland is limited. We conducted two field experiments with 7 levels of N (from 0.5 g to 24 g N ∙ m^−2^ yr^−1^) and P (from 0.05 g to 3.2 g P ∙ m^−2^ yr^−1^) additions in a desert grassland of Kunlun Mountain in the northwest of China to investigate the effects of these addition rates on the N and P stoichiometry of the dominant grass species *Seriphidium korovinii*. Nitrogen and P additions both affected plant stoichiometry. N addition suppressed P concentrations, whereas P addition had no effect on plant N concentrations. The N:P ratios of green aboveground biomass (AGB) were positively correlated with N addition ranging from 14.73 to 29.08, whereas those for P additions decreased ranging from 14.73 to 8.29. N concentrations were positively correlated with soil available N:P ratios, whereas, P concentrations were negatively correlated with soil availably N:P. Our results suggest that chemistry and stoichiometry of *S. korovinii* was directly affected by soil nutrient availability. Soil N availability affects *S. korovinii* stoichiometry to a greater extent that does soil P availability in this ecosystem. These findings suggest that N-deposition could affect the stoichiometry of this desert grassland ecosystem, and thereby potentially alter litter decomposition, plant community composition, nutrient cycling, and food-web dynamics of these desert ecosystems.

## Introduction

Ecological stoichiometry mainly investigates the balance of C, N, P in ecological processes^1,2^. Plant leaves C:N:P stoichiometry controls ecosystem processes through its effects on plant growth^3^, patterns of herbivory^4^, litter decomposition^5–7^, microbial interactions and community dynamics^8^, and nutrient cycling^9^. Moreover, ecological stoichiometry is sensitive with increased N deposition^10^, fire^11^, precipitation^12^, elevated CO_2_^13^, land use change^14^ and the interactions among these factors^12,13,15^. Consequently, plant C:N:P stoichiometry is critical to help us clarify the responses of biogeochemical and ecological patterns and process to global change.

Plant ecological stoichiometry is closely related to metabolic conditions^16^. N and P are tightly coupled between soil and plant nutrient demands^17,18^. Leaf C:N and C:P reflect the ability of plant in assimilating C under N or P accumulation^19^, and are often correlate to plant relative growth rate^20^. However, the relationship of plant relative growth rate and N:P ratios is conflicting in different nutrient status of plant. For instance, plant relative growth rates are positively correlated with N:P ratios under N limitation but are negatively correlated under P limitation^20^. Foliar N:P ratios have been used as indicator to assess restriction nutrient in terrestrial ecosystems^21^. For example, N:P mass ratio <10 is supposed to N limitation, while N:P mass ratio >20 corresponds to P limitation for terrestrial plants. However, only a very small of fertilization studies have been conducted in desert grasslands. Drenovsky and Richards (2004) reported that critical N:P values are unsuitable to indicate the N and P limitations of desert shrublands because of species-specific critical N:P values^9^. Thus, more studies are needed for a general conclusion in terms of plant ecological stoichiometry response to nutrient availability.

Plant N and P concentrations are directly influenced by soil N and P availability. In grassland ecosystem, N fertilization typically increase foliar N concentrations, while reduce C:N ratios^10^, and further affect litter decomposition rates^22^. However, previous studies have reported positive^18,23^, negative^24^ and no^24^ effects of N addition on plant P concentrations and N:P ratios, due to variation in species-specific response patterns of plants, experimental duration in different studies, and the amount of N addition. Rarely studies have reported that N and P concentrations presents various change trends with P addition rates^18,25^. Given the conflicting results of these studies, extra evidence from various ecosystem types are needed to distinguish general patterns in plant ecological stoichiometry responses to nutrient addition.

Nutrient additions such as N and P are a common strategy to improve grassland productivity and restore the degraded grasslands^26^. Besides the artificial additions by human activities, the natural processes such as N deposition and sand storm can also have significant effect on soil nutrient availability. This is especially true for the desert grassland on the northern slope of Kunlun Mountain in northwest China. Frequently occurring northwest wind can bring abundant sandy soil rich in P to the ecosystem^27^. The local farmers also frequently apply N and P fertilizers to ensure the productivity for animal grazing. These extra nutrient could have major effect on plant ecological stoichiometry^12,18^. Hence, a better understanding of plant ecological stoichiometric responses to concurrent alterations in N and P availability is critical for projection of nutrient cycling dynamics under future global change.

The objective of this study was to assess the effects of artificial N and P additions on plant C, N, and P concentrations, and their ratios under desert ecosystem types. Hence, field experiments with contrasting N and P addition rates were established at desert grassland in Kunlun Mountain with the following hypotheses: (1) grass grown in N-enriched soil would have lower C:N and higher N:P, and higher C:P in senesced tissues result from an expected increase in P resorption in response to N addition; (2) P addition can reduce C:N in senesced tissues due to an increase in N resorption, and lower N:P and C:P ratios; and (3) N addition will result in the P limitation or increase P demand, as evidenced by foliar N:P stoichiometry, and P addition will result in N limitation or increase N demand.

## Results

**Figure 1:**
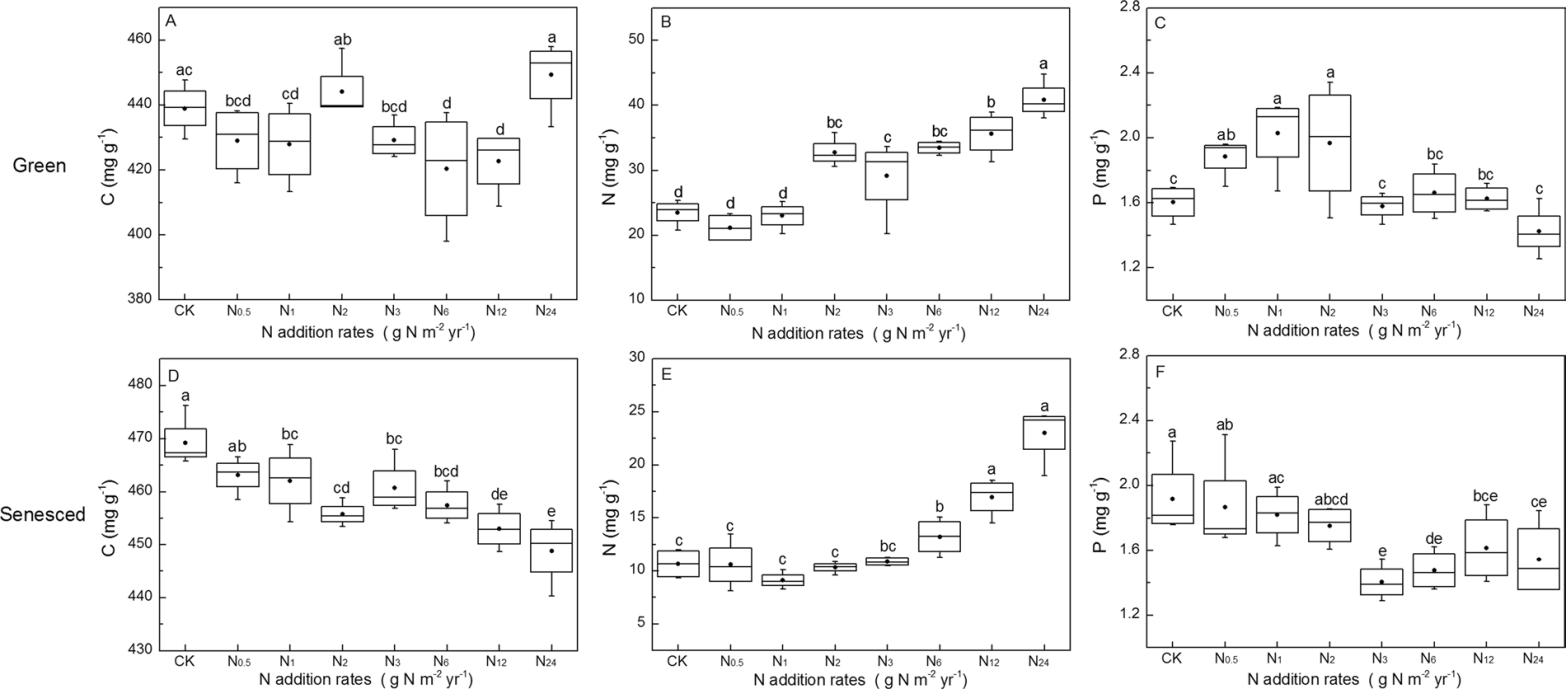
Changes of C, N, P concentrations of *S. korovinii* green and senesced tissues aboveground biomass under various N addition rates. Each box represents the interquartile range, with median indicated. Whiskers represent the 10th and 90th quartiles, black full circles indicate mean. The same letter indicates no significantly different (P < 0.05).

**Figure 2:**
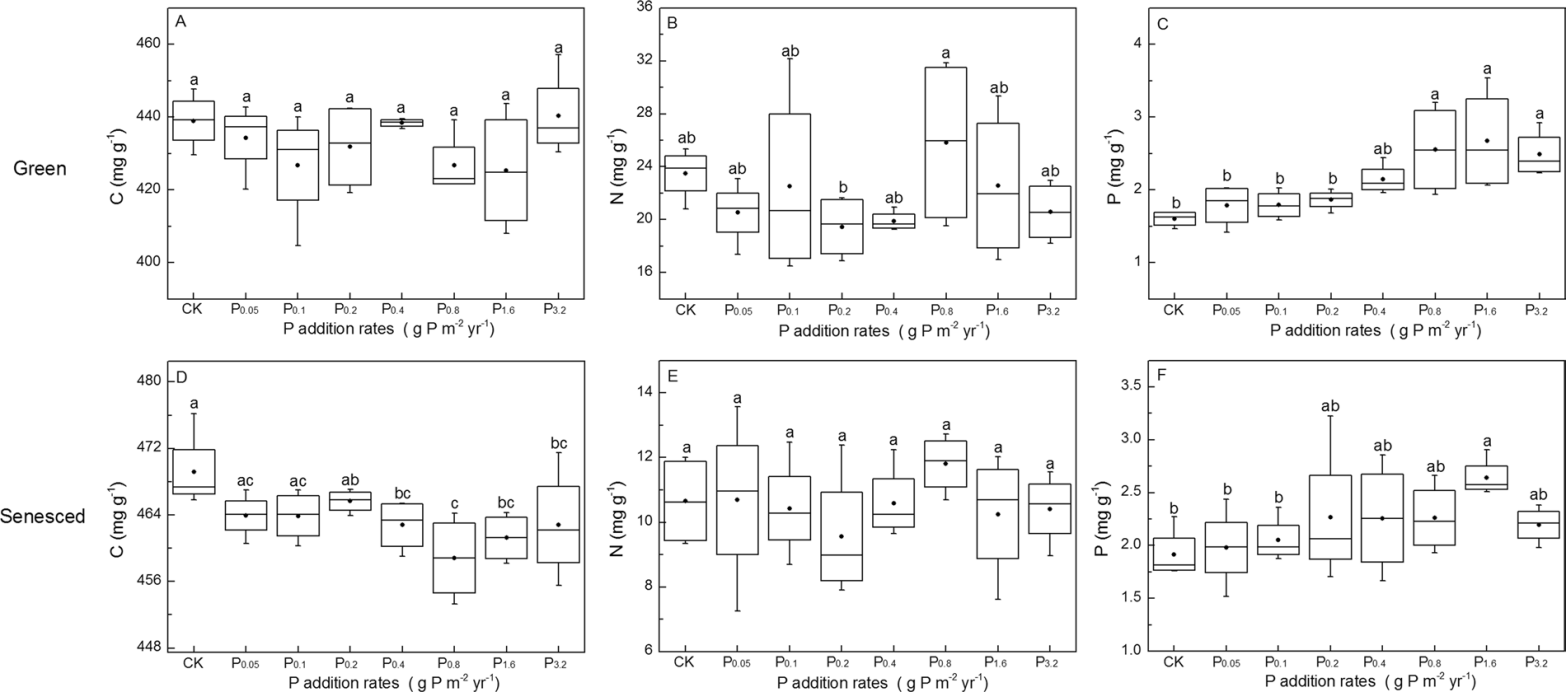
Changes of C, N, P concentrations of *S. korovinii* green and senesced tissues aboveground biomass under various P addition rates. Each box represents the interquartile range, with median indicated. Whiskers represent the 10th and 90th quartiles, black full circles indicate mean. The same letter indicates no significantly different (P < 0.05).

**Figure 3:**
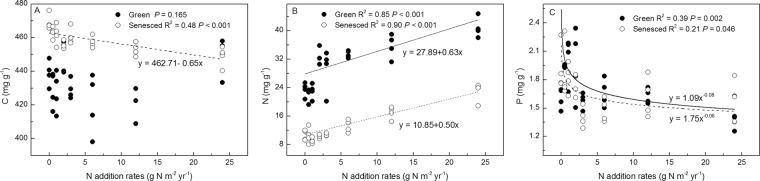
Relationship between *S. korovinii* biomass C(**A**), N(**B**), P(**C**) concentration of green and senesced aboveground biomass and N addition rates. Solid lines are the linear or nonlinear regression models between C, N, P concentration of green aboveground biomass and N addition rates. Dashed lines are the linear or nonlinear regression models between C, N, P concentration of senesced aboveground biomass and N addition rates.

**Figure 4:**
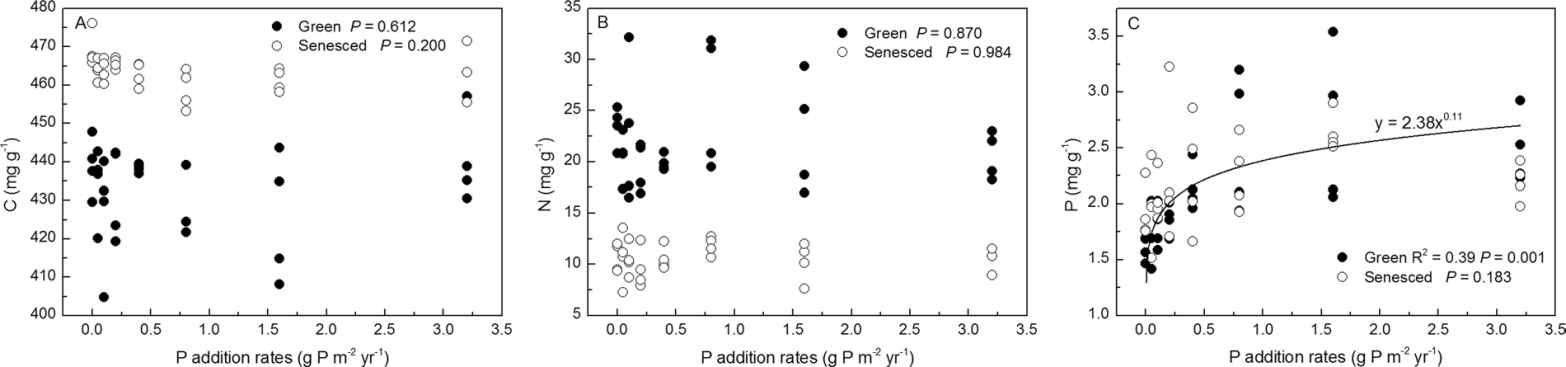
Relationship between *S. korovinii* biomass C(**A**), N(**B**), P(**C**) concentration of green and senesced aboveground biomass and P addition rates. Solid lines are the nonlinear regression models between C, N, P concentration of green aboveground biomass and P addition rates.

C, N, P concentrations of both green and senesced AGB were affected by short-term N and P additions (Figs. 1 and 2). The C concentration of senesced AGB were negatively correlated with N addition rates (*P* < 0.001, *R*^2^ = 0.48), whereas those of green tissues were not significantly affected (*P* = 0.165). Nitrogen concentrations in both green and senesced AGB were positively (*P* < 0.001) correlated with N addition rates with *R*2 of 0.85 and 0.90, respectively. By contrast, the P concentrations in green and senesced AGB were negatively (*P* < 0.05) correlated with N addition rates with *R*2 of 0.39 and 0.21, respectively (Fig. 3). P addition rates had no effect on C and N concentrations of green and senesced AGB. The P concentration of green AGB were positively correlated with P addition rates (*P* = 0.001, *R*^2^ = 0.39), whereas those of senesced tissues had no significant changes (*P* = 0.183) (Fig. 4).

**Figure 5:**
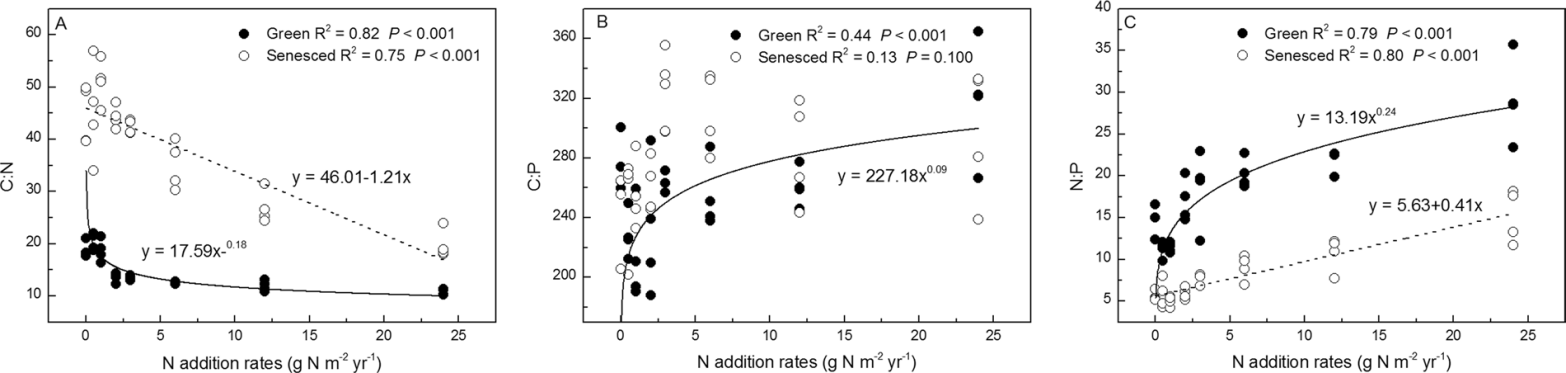
Relationship between ratios of C, N, P of *S. korovinii* green and senesced aboveground biomass and N addition rates. Solid lines are the nonlinear regression models between ratios of C, N, P of green aboveground biomass and N addition rates. Dashed lines are the linear or nonlinear regression models between ratios of C, N, P of senesced aboveground biomass and N addition rates.

**Figure 6:**
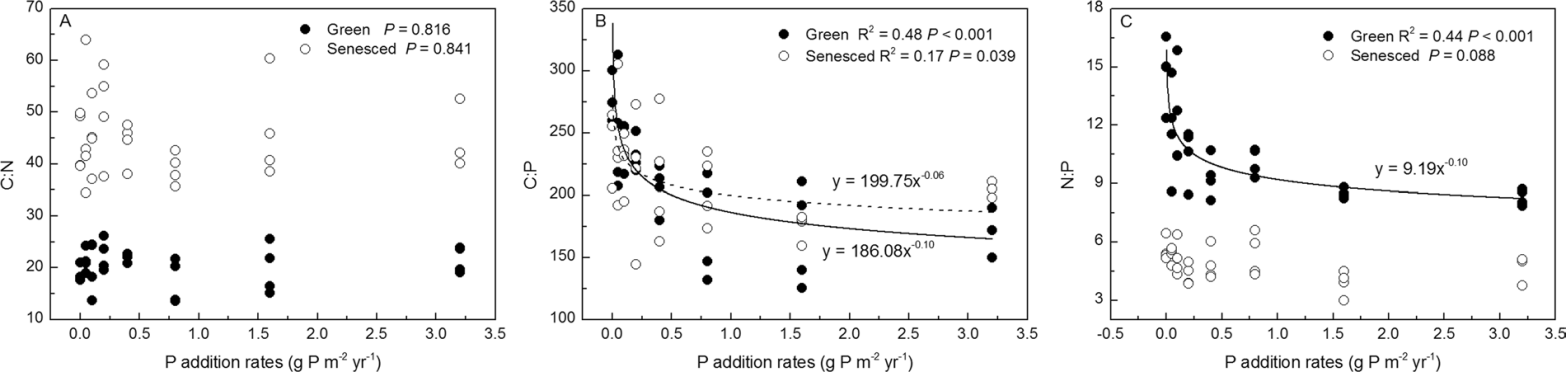
Relationship between ratios of C, N, P of *S. korovinii* green and senesced aboveground biomass and P addition rates. Solid lines are the nonlinear regression models between ratios of C, N, P of green aboveground biomass and P addition rates. Dashed lines are the nonlinear regression models between ratios of C, N, P of senesced aboveground biomass and P addition rates.

The C:N ratios of green and senesced AGB were negatively correlated with N addition rates (*P* < 0.001, *R*^2^ = 0.82 and 0.75, respectively), whereas the C:P and N:P ratios were positively correlated with N addition rates (*P* < 0.001), except for C:P ratios of senesced AGB (Fig. 5). Both green and senesced AGB C:N ratios had no significant changes with increasing P application (*P* = 0.816, *P* = 0.841), whereas the C:P ratios of green and senesced AGB were negatively correlated with P addition rates (*P* < 0.05). The N:P ratios of green AGB were negatively correlated with N addition rates (*P* < 0.001, *R*^2^ = 0.44), whereas those of senesced tissues had no significant changes (*P* = 0.088) (Fig. 6).

**Figure 7:**
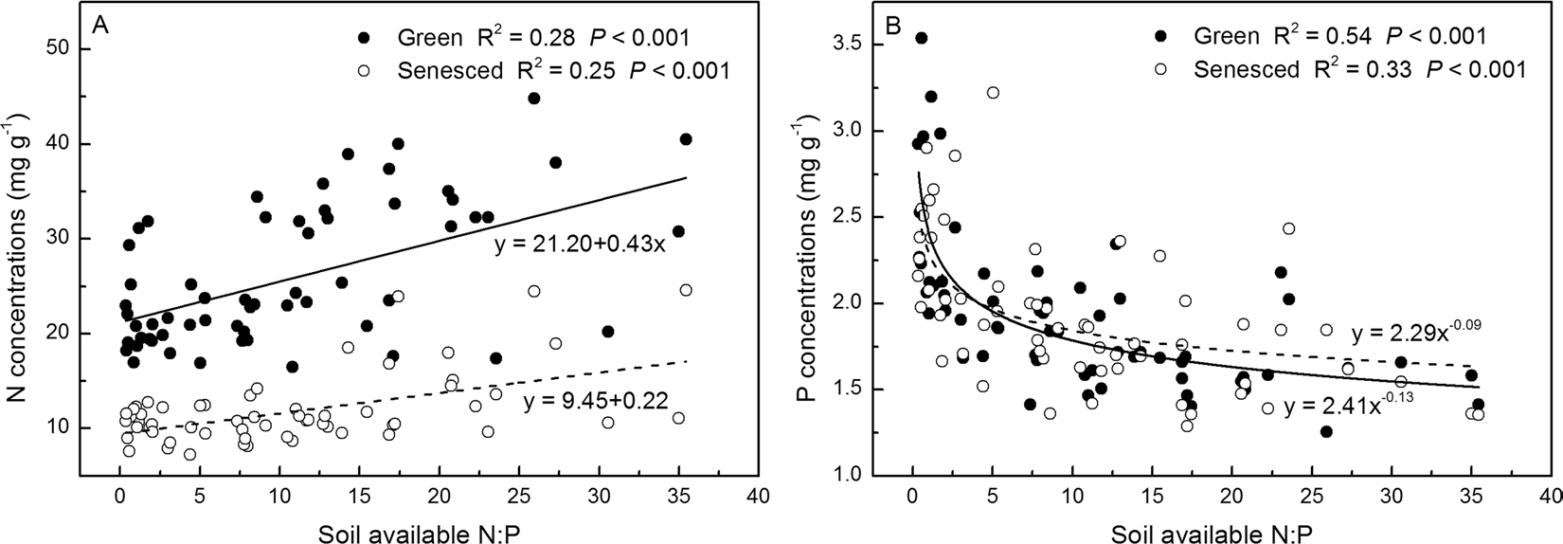
Relationships between soil available N:P and *S. korovinii* biomass N (**A**), P (**B**) concentrations of green and senesced aboveground biomass under N and P addition. Solid lines are the linear or nonlinear regression models between N, P concentration of green aboveground biomass and soil available N:P. Dashed lines are the linear or nonlinear regression models between N, P concentration of senesced aboveground biomass and soil available N:P.

**Figure 8:**
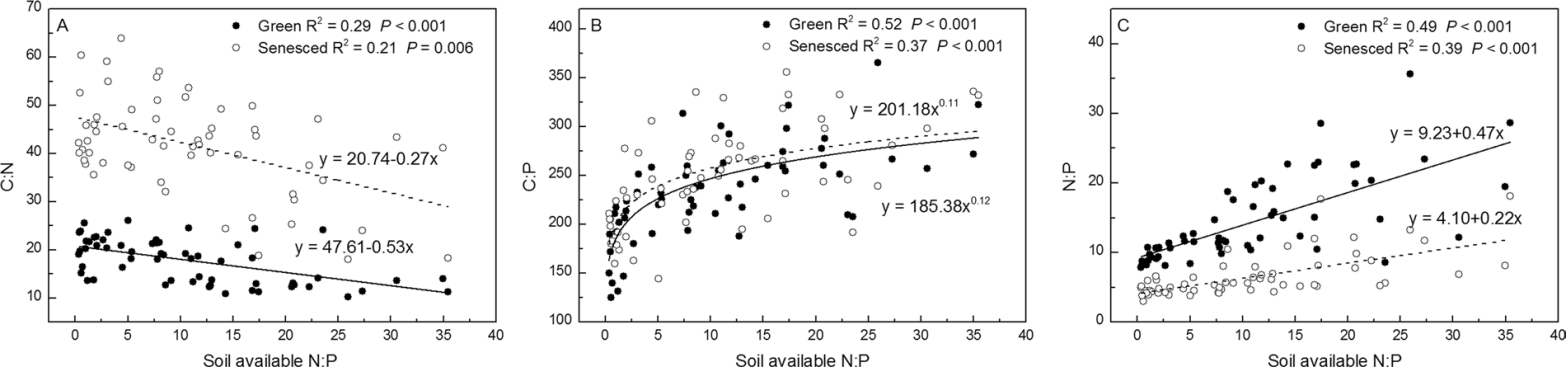
Relationships between soil available N:P and ratios of C, N, P of *S. korovinii* green and senesced aboveground biomass under N and P addition. Solid lines are the linear or nonlinear regression models between ratios of C, N, P of *S. korovinii* green aboveground biomass and soil available N:P. Dashed lines are the linear or nonlinear regression models between ratios of C, N, P of *S. korovinii* senesced aboveground biomass and soil available N:P.

N concentrations and C:P and N:P ratios of both green and senesced AGB correlated positively with soil available N:P, whereas P concentrations and C:N ratios negatively correlated with soil available N:P (*P* < 0.001) (Figs. 7 and 8).

## Discussion

Our results clearly show that the plant AGB C:N:P stoichiometry of the dominant desert grass species was affected by short-term N and P additions. Our results are consistent with the hypotheses that C:N ratios would decline and the ratios of N:P and C:P would increase with increasing N addition. However, our hypotheses that the ratios of C:N, C:P and N:P would decline with increasing P addition were not fully supported. In fact, C:N and N:P ratios in both green and senesced AGB were generally not affected by P addition (Fig. 5, *P* > 0.05). These results suggest that N availability affects *S. korovinii* stoichiometry more than P availability in this study system. Moreover, the plant stoichiometry was strongly related to soil available N:P (Figs. 6 and 7, *P* < 0.05).

N and P concentrations correlational relationship is weak^21^. N addition would enrich N and increase P limitation and demand for plant growth^28–31^, affect the plant P concentration after N fertilization^17,32^. However, positive or no effects of N addition on plant P concentrations have also been reported previously. For instance, long-term N addition does not affect the P concentrations of two bryophytes grown in acidic grassland^33^. Lü *et al*. (2013) reported positive relationships between the rates of N addition and P concentrations in both green and senesced leaves of grassland grown in a semiarid region. Variation in the patterns observed between increasing N input effects on plant could be because that N addition stimulates root-surface phosphomonoesterase activities^30^, enhances P conservation, and accelerates P cycling rates^34^.

The geometric means of leaf N, P, and N:P ratio for the 753 species in China were 18.6 and 1.21 mg g^−1^ and 14.4^35^, respectively, and the global geometric means were 18.3 and 1.42 mg g^−1^ and 11.8^36^, respectively. Our results show that the N and P concentrations of the green AGB of *S. korovinii* without N inputs were 23.5 mg g^−1^ and 1.60 mg g^−1^, respectively, with an N:P ratio of 14.73 (Fig. 2). These results largely differ with our previous study with N and P concentrations of 9.87 and 2.98 mg g^−1^, respectively and an N:P ratio of 3.31, suggesting N limitation^23^. We suspect that the sampling time, and the water conditions caused by annual precipitation patterns, leading to these large variations^10,37^. As expected, N addition significantly increased N concentrations of both green and senesced AGB. By contrast, P concentrations in plant decrease with increasing N addition rates (Fig. 2, *P* < 0.05). Consequently, lower C:N ratios and higher C:P ratios were found at high N addition rates (Fig. 4), being consistent with other studies^10^. These results may account for lower N resorption efficiencies and higher P resorption efficiencies after N addition^33,38^. Moreover, N addition would increase the plant productivity, resulting in a growth dilution effect of P concentrations^39^. Accordingly, N inputs reduced plant dependence on internal N recycling by obtaining more N from their environment, as indicated by the higher N concentration of senesced AGB, which increased the amount of N returned to the soil. Whereas, N inputs increased P recycling, as indicated by the low P concentration of both green and senesced tissues. Hence, the internal N and P recycling of plant were affected by N fertilization.

Enriched soil N and P availability would be expected with higher plant concentrations of N and P. Moreover, P input would significantly decline the P resorption efficiency and more P remained in senesced leaves. For instance, P concentrations of both green and senesced leaves increased with increasing P addition rates in alpine grassland^18^. However, results show that the P concentrations of green AGB were positively correlated with P addition rates (Fig. 3C, *P* = 0.001), whereas those of senesced were not significantly affected (Fig. 3C, *P* = 0.183). These results would increase P resorption efficiency, which could be found in other reports^18^. Interestingly, results also show that P concentrations of green AGB had no changes and even lower than that of senesced tissues (Figs. 2 and 3, *P* > 0.05), suggesting that P addition increased plant P concentrations in the litterfall which means more P was being recycled through the plant-soil system, and plants were being less conservative with their P. Hence, the responses of P concentrations in plant in response to P addition are complex and further studies with plants under species-specific and environments-specific require a more general conclusion. Furthermore, N concentrations were not significantly affected by P addition, which was consistent with results of the P fertilization experiment of *Leymus chinensis* and *Stipa grandis* in a semi-arid grassland^25^. Taken together, the N and P concentrations of AGB are sensitive to N availability but not in P in our study.

Interestingly, C concentrations of senesced AGB were negatively correlated with N addition rates (Fig. 2A, *P* < 0.001). These results suggest that N addition may potentially affect the carbon pools of plant, although plant biomass was not evaluated. Plant stoichiometry is highly sensitive to soil N addition rates (Figs. 2–5) and soil inorganic N (Figs. S1–4) than P addition rates and soil available P. Plant stoichiometry are correlative with soil available N:P ratios (Figs. 6 and 7, *P* < 0.05). Moreover, the slope of the line between soil available N:P ratios and plant P concentrations of AGB is steep at low soil available N:P ratios and then becomes flat at very high soil available N:P ratios (Fig. 6), given that the low values of N:P ratios of AGB at low soil available N:P ratios and high values of N:P ratios of AGB at high soil available N:P ratios, and plant relative growth rates are positively correlated with N:P ratios under N limitation but are negatively correlated under P limitation^20^. These results suggest that N:P ratios were also regulated by the stoichiometry of plant directly affected by soil nutrient availability. Soil N availability affects plant stoichiometry to a greater extent that does soil P availability in this ecosystem. Hence, ongoing N deposition could significantly modify the stoichiometry of these desert ecosystems, thereby potentially alter litter decomposition, plant community composition, nutrient cycling, and food-web dynamics.

In conclusion, this study demonstrate that both N and P addition affect plant stoichiometry. Further, N and P addition show different effects, where N addition suppresses P concentration but P addition has no effect on plant N concentration. N:P ratios were regulated by soil nutrient availability through the stoichiometry of plant. Soil N availability affects plant stoichiometry to a greater extent that does soil P availability in this ecosystem. N deposition would affect the stoichiometry, and thereby potentially altering litter decomposition, plant community composition, nutrient cycling, food-web dynamics of these desert ecosystems. This study provides detailed insights about plant stoichiometry in response to short-term nutrient additions and suggests that N and P concentrations in soil play an important role in mediating plant stoichiometry responses to nutrient addition in desert grassland.

## Materials and Methods

### Study area and experimental design

This study was conducted in the desert grassland (80°43′38″E, 36°22′54″N) of Kunlun Mountain in northwest China. The study area had been fenced since 2009 to prevent the grazing of large animals. Detailed information about the study site has been reported in a previous study^23^. Briefly, the mean annual temperature was 3 °C. The mean annual precipitation varies from 60 to 150 mm, and more than 85% of the total precipitation occurs in the growing season from May to October. The soil type is brown desert soil, and the vegetation type is desert grasses. The perennial grass *S. korovinii* is the dominant plant species, which represent almost 90% of the total vegetation.

Early May of 2017, field experiments as N and P addition were established in flat land (<2% slope) with totally new experimental plots compare with our previously study^23^. This study was a multifactorial experiment, which considered N and P addition levels as two nutrient factors. A total 15 treatments was conducted including 7 addition levels for N (0.5–24 g N ∙ m^−2^ yr^−1^) and P (0.05–3.2 g P ∙ m^−2^ yr^−1^), respectively, as well as an unfertilized control shared by both N and P factors. Seven N rates of 0, 0.5, 1, 2, 3, 6, 12, and 24 g N ∙ m^−2^ yr^−1^ of urea and seven P rates of 0, 0.05, 0.1, 0.2, 0.4, 0.8, 1.6, and 3.2 g P ∙ m^−2^ yr^−1^ of KH_2_PO_4_ were applied. The determination of these application rates was based on a previous study in grassland from Bayanbulak, Xinjiang, China^40^. Treatments were laid out in a randomized block design with four replicate plots that are 3 m × 2 m size. A total of 60 plots were used. All plots were separated from each other by a 1 m buffer area to prevent fertilizer movement between the experimental plots. In May of 2017, all fertilizers in each experiment were thoroughly mixed with soil and broadcasted to the plot surface during rainy days.

### Plant and soil sampling and measurements

On 10 to 12 July 2017, the AGB of *S. korovinii* in the subplot (1 m × 1 m) of each plot was collected. After the removal of impurities, the AGB samples were oven-dried at 75 °C for 48 h, and then ground to pass a 1 mm sieve for elemental analysis. On October 28 to 30 2017, the senescent AGB of *S. korovinii* in the subplot of each plot also were collected, dried, and ground. C and N concentrations in plant tissues were analyzed with a CN elemental analyser (Eurovector, Milan, Italy). Phosphorus concentration was determined by persulfate oxidation followed by colorimetric analysis. Mass ratios of C:N, C:P and N:P were used to facilitate comparisons with previous studies^10,21^.

On 13 July 2017, four soil samples (0 cm to 10 cm) were randomly collected using a 2 cm-diameter soil auger from each plot, and combined as a single composite sample. All soil samples were sieved through a 2-mm mesh to remove their roots and impurities. The inorganic N in the soil was measured with a flow injection autoanalyzer (FIAstar 5000, Foss Tecator, Denmark). The available P concentrations in the soil were determined by the ammonium molybdate method. The concentrations of inorganic N and available P in the soil were based on the dry soil weight, which was determined by drying the soil at 105 °C for 48 h^18^.

### Statistical analysis

Data were tested for normality using the Kolmogorov–Smirnov test and for equality of error variance using Levene’s test. One-way ANOVA was performed to examine the nutrient (N and P) addition rates effect on plant stoichiometry. Least significant difference (LSD) post-hoc tests were conducted to determine the differences between the individual treatments. Regression models (y = ax + b or y = ax^b^) were used to determine N and P application rate and various responses. Moreover, correlation analyses were used to examine the general linear regression and nonlinear regression. All statistical analyses were performed with SPSS version 19.0 (SPSS Inc., Chicago, IL, USA).

## Supplementary information

Supplementary Figures

## References

[1] Elser JJ, Hamilton A (2007). Stoichiometry and the New Biology: The Future Is Now. PLOS Biol..

[2] Sterner, R. W. & Elser, J. J. *Ecological stoichiometry: the biology of elements from moleculas to the biosphere*. *Prinecton University Press* (2002).

[3] Zhang B (2018). Groundwater Depth Affects Phosphorus But Not Carbon and Nitrogen Concentrations of a Desert Phreatophyte in Northwest China. Front. Plant Sci..

[4] Daufresne T, Loreau M (2010). Plant-herbivore interactions and ecological stoichiometry: when do herbivores determine plant nutrient limitation?. Ecol. Let..

[5] Hättenschwiler S, Jørgensen HB (2010). Carbon quality rather than stoichiometry controls litter decomposition in a tropical rain forest. J. Ecol..

[6] Manzoni S, Trofymow JA, Jackson RB, Porporato A (2010). Stoichiometric controls on carbon, nitrogen, and phosphorus dynamics in decomposing litter. Ecol. Monogr..

[7] Güsewell S, Gessner MO (2009). N: P ratios influence litter decomposition and colonization by fungi and bacteria in microcosms. Funct. Ecol..

[8] Zechmeister-Boltenstern S (2015). The application of ecological stoichiometry to plant–microbial–soil organic matter transformations. Ecol. Monogr..

[9] McGroddy ME, Daufresne T, Hedin LO (2004). Scaling of C:N:P stoichiometry in forests worldwide: Implications of terrestrial redfield‐type ratios. Ecology.

[10] Drenovsky RE, Richards JH (2004). Critical N:P values: Predicting nutrient deficiencies in desert shrublands. Plant Soil.

[11] Cui Q, Lü XT, Wang QB, Han XG (2010). Nitrogen fertilization and fire act independently on foliar stoichiometry in a temperate steppe. Plant Soil..

[12] Lu XT, Kong DL, Pan QM, Simmons ME, Han XG (2012). Nitrogen and water availability interact to affect leaf stoichiometry in a semi-arid grassland. Oecologia.

[13] Knops JMH, Naeem S, Reich PB (2007). The impact of elevated CO2, increased nitrogen availability and biodiversity on plant tissue quality and decomposition. Global Change Biol..

[14] Zhao FZ (2015). Land use change influences soil C, N, and P stoichiometry under ‘grain-to-green program’ in china. Sci. Rep..

[15] Novotny AM (2007). Stoichiometric response of nitrogen-fixing and non-fixing dicots to manipulations of CO2, nitrogen, and diversity. Oecologia.

[16] Rong Q (2015). Leaf carbon, nitrogen and phosphorus stoichiometry of Tamarix chinensis Lour. in the Laizhou Bay coastal wetland, China. Ecol. Eng..

[17] Menge DNL, Field CB (2007). Simulated global changes alter phosphorus demand in annual grassland. Global Change Biol..

[18] Li L (2016). Nitrogen (N) and phosphorus (P) resorption of two dominant alpine perennial grass species in response to contrasting N and P availability. Environ. Exp. Bot..

[19] Wang, Z., Lu, J., Yang, M., Yang, H. & Zhang, Q. Stoichiometric Characteristics of Carbon, Nitrogen, and Phosphorus in Leaves of Differently Aged Lucerne (Medicago sativa) Stands. *Front. Plant Sci*. **6** (2015). 10.3389/fpls.2015.01062 PMC4673304 26697029

[20] Yu Q (2012). Testing the Growth Rate Hypothesis in Vascular Plants with Above- and Below-Ground Biomass. PLOS ONE.

[21] Güsewell SN (2004). P ratios in terrestrial plants: variation and functional significance. New Phytol..

[22] Mo J, Brown S, Xue J, Fang Y, Li Z (2006). Response of Litter Decomposition to Simulated N Deposition in Disturbed, Rehabilitated and Mature Forests in Subtropical China. Plant Soil.

[23] Li L (2017). Stoichiometry in aboveground and fine roots of Seriphidium korovinii in desert grassland in response to artificial nitrogen addition. J. Plant Res..

[24] Huang W (2012). Effects of elevated carbon dioxide and nitrogen addition on foliar stoichiometry of nitrogen and phosphorus of five tree species in subtropical model forest ecosystems. Environ.Pollut..

[25] Lü X, Reed SC, Yu Q, Han X (2016). Nutrient resorption helps drive intra-specific coupling of foliar nitrogen and phosphorus under nutrient-enriched conditions. Plant Soil.

[26] Conant RT, Paustian K, Elliott ET (2001). grassland management and conversion into grassland: effects on soil carbon. Ecol. Appl..

[27] Okin GS, Mahowald N, Chadwick OA, Artaxo P (2004). Impact of desert dust on the biogeochemistry of phosphorus in terrestrial ecosystems. Global Biogeochem. Cy..

[28] Vitousek PM, Porder S, Houlton BZ, Chadwick OA (2010). Terrestrial phosphorus limitation: mechanisms, implications, and nitrogen–phosphorus interactions. Ecol. Appl..

[29] Phuyal M, Artz RRE, Sheppard L, Leith ID, Johnson D (2008). Long-term nitrogen deposition increases phosphorus limitation of bryophytes in an ombrotrophic bog. Plant Ecol..

[30] Phoenix GK (2004). Simulated pollutant nitrogen deposition increases P demand and enhances root‐surface phosphatase activities of three plant functional types in a calcareous grassland. New Phytol..

[31] Tao L, Hunter MD (2012). Does anthropogenic nitrogen deposition induce phosphorus limitation in herbivorous insects?. Global Change Biol..

[32] Heerwaarden LMV, Toet S, Aerts R (2003). Nitrogen and phosphorus resorption efficiency and proficiency in six sub‐arctic bog species after 4 years of nitrogen fertilization. J. Ecol..

[33] Arróniz‐Crespo M, Leake JR, Horton P, Phoenix GK (2008). Bryophyte physiological responses to, and recovery from, long‐term nitrogen deposition and phosphorus fertilisation in acidic grassland. New Phytol..

[34] Marklein AR, Houlton BZ (2012). Nitrogen inputs accelerate phosphorus cycling rates across a wide variety of terrestrial ecosystems. New Phytol..

[35] Han W, Fang J, Guo D, Zhang Y (2005). Leaf nitrogen and phosphorus stoichiometry across 753 terrestrial plant species in China. New Phytol..

[36] Reich PB, Oleksyn J (2004). Global patterns of plant leaf N and P in relation to temperature and latitude. P. NATL. ACAD. SCI. USA.

[37] Zhang H (2013). Sampling Date, Leaf Age and Root Size: Implications for the Study of Plant C:N:P Stoichiometry. PLOS ONE.

[38] Li L, Zeng D-H, Mao R (2012). & Z-Y, Y. Nitrogen and phosphorus resorption of Artemisia scoparia, Chenopodium acuminatum, Cannabis sativa, and Phragmites communis under nitrogen and phosphorus additions in a semiarid grassland, China. Plant Soil Environ..

[39] Perring MP, Hedin LO, Levin SA, McGroddy M, de Mazancourt C (2008). Increased plant growth from nitrogen addition should conserve phosphorus in terrestrial ecosystems. P. NATL. ACAD. SCI. USA.

[40] Yue P (2016). A five-year study of the impact of nitrogen addition on methane uptake in alpine grassland. Sci. Rep..

