# Supplementary material for: Nitrogen and phosphorus addition differentially affect plant ecological stoichiometry in desert grassland
Source: Sci Rep. 2019 Dec 10;9:18673. doi: 10.1038/s41598-019-55275-8 (PMC6904472; doi:10.1038/s41598-019-55275-8)
Supplement: Supplementary file 1 — Supplementary Figures [file 41598_2019_55275_MOESM1_ESM.docx]

**Supplement information:**

Manuscript title: Nitrogen and phosphorus addition differentially affect plant ecological stoichiometry in desert grassland

Author list: Lei Li, Bo Liu, Xiaopeng Gao, Xiangyi Li, Chengdao Li


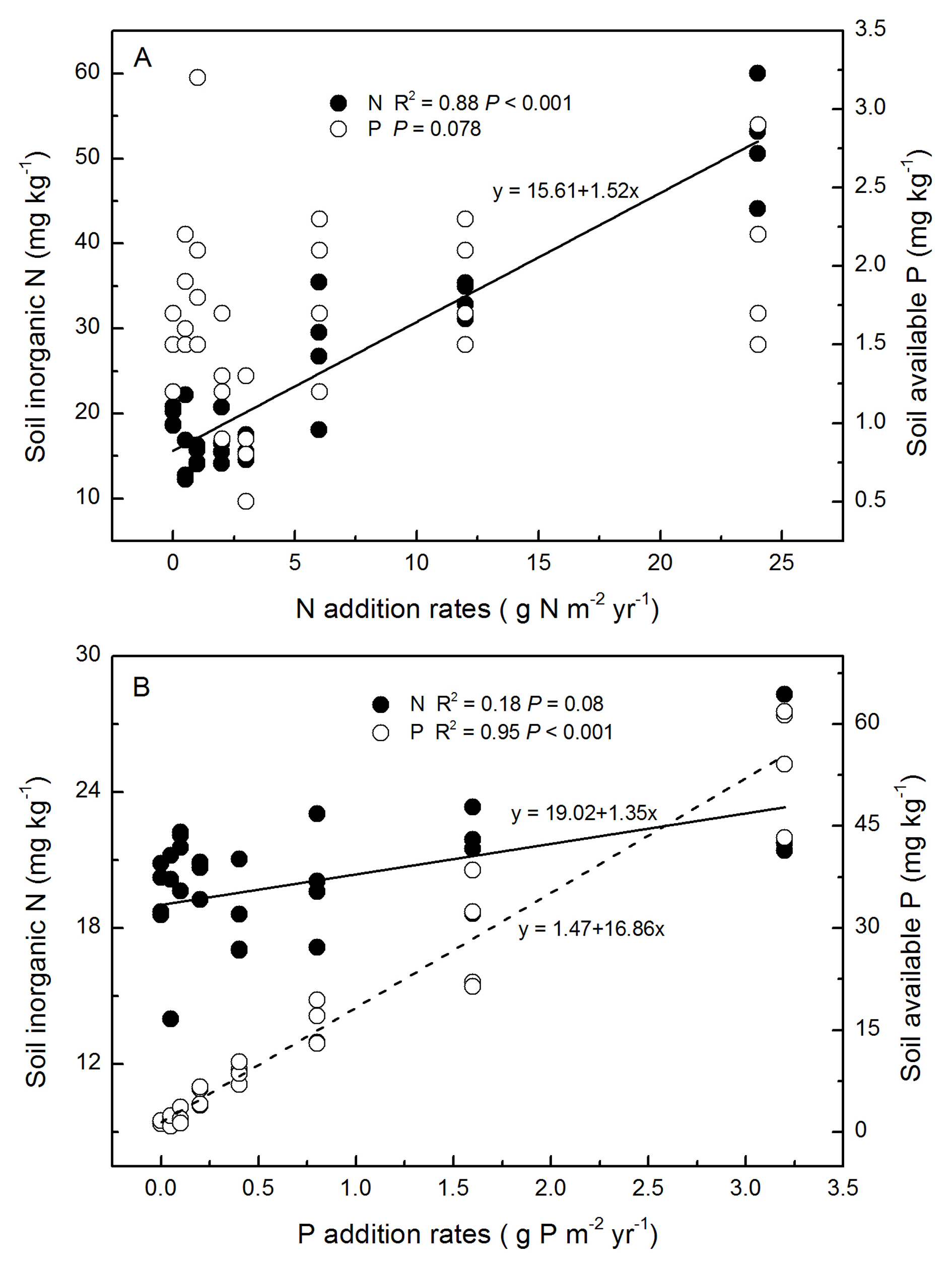


Fig. 1 Relationships between N and P addition rates and soil available N and P

**
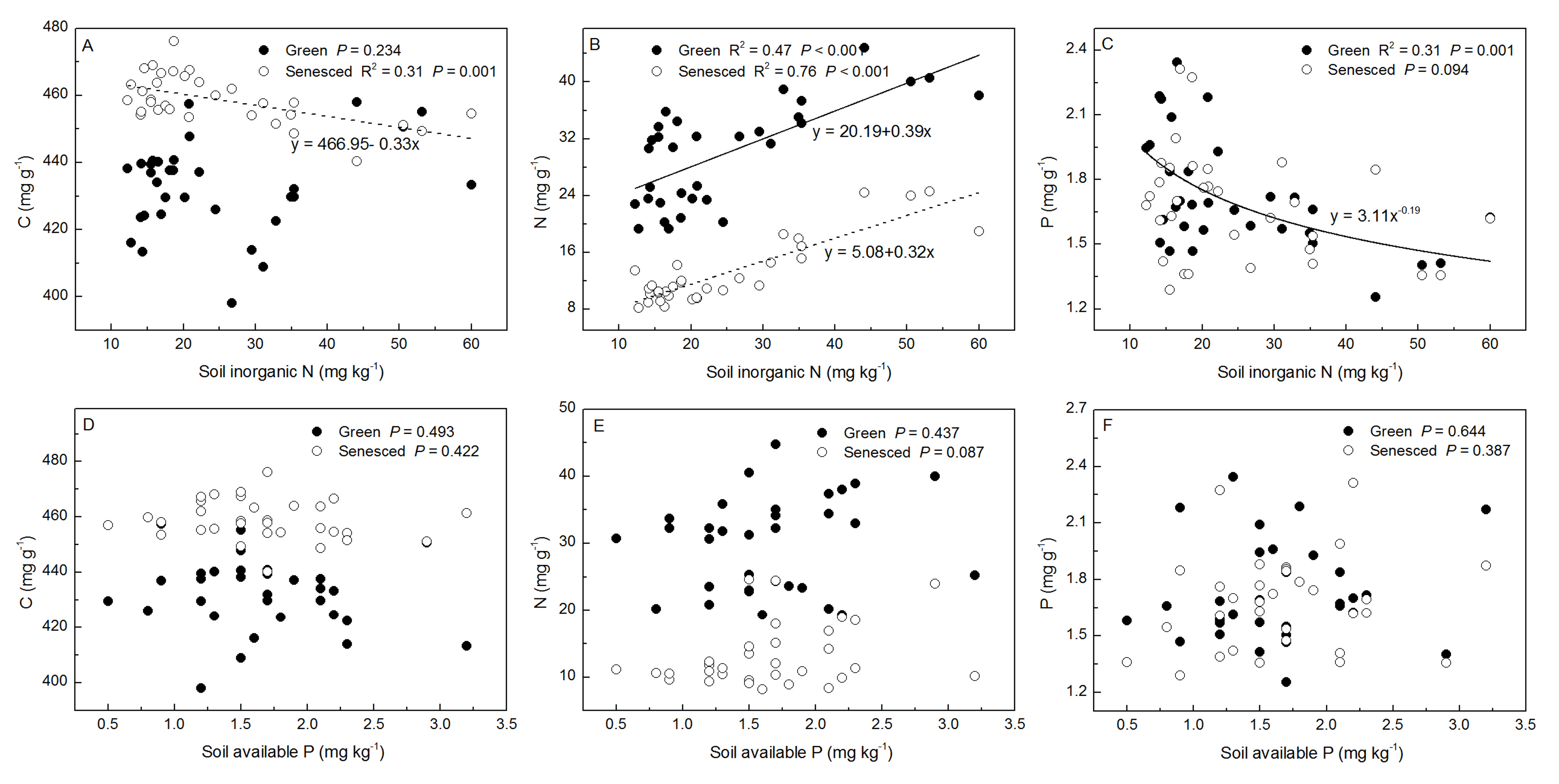
**

Fig. S2 Relationship between C(A), N(B), P(C) concentration of green and senesced aboveground biomass and soil available nutrient under N additions.


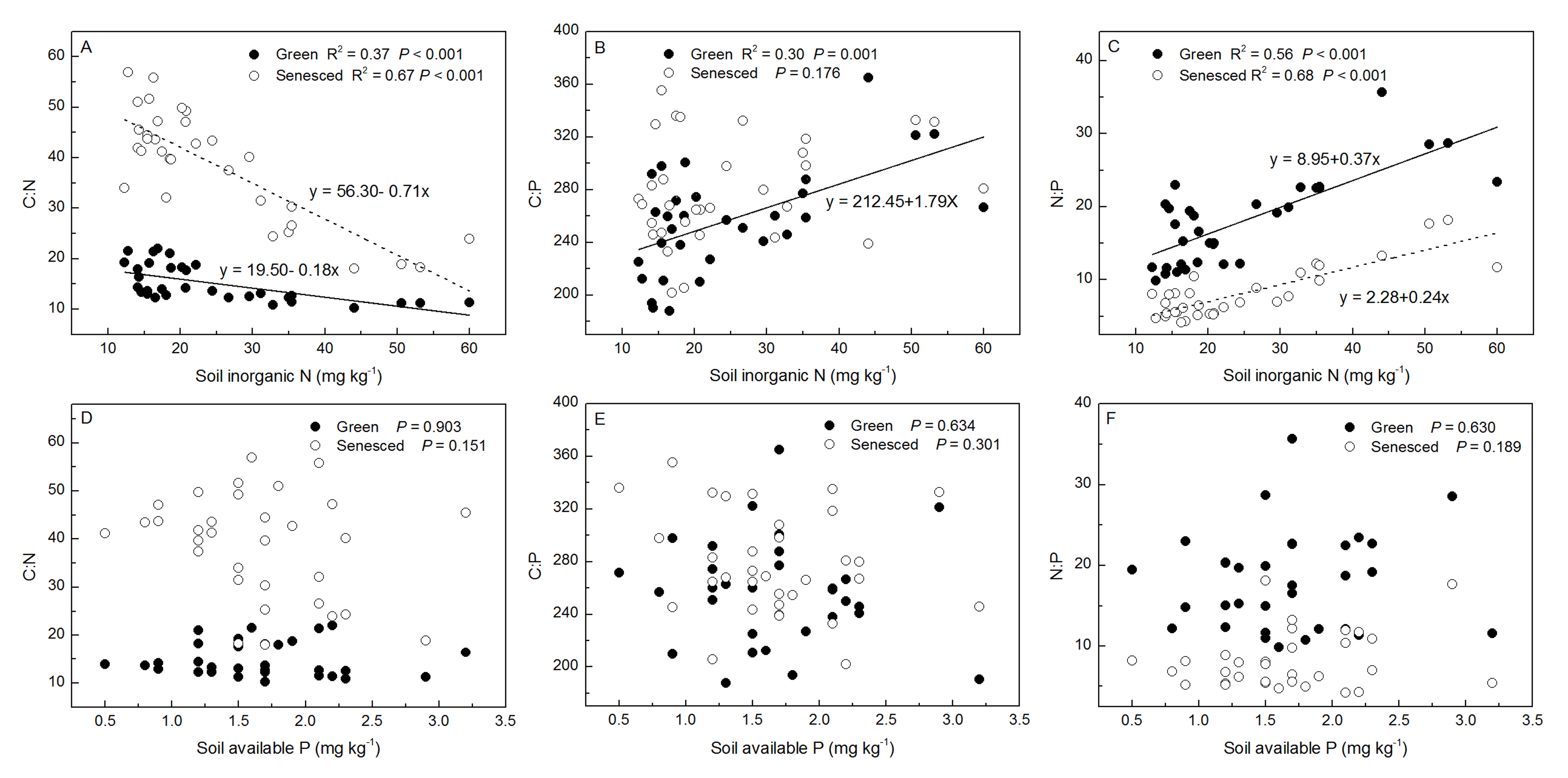


Fig. S3 Relationship between ratios of C, N, P of green and senesced aboveground biomass and soil available nutrient under N additions.


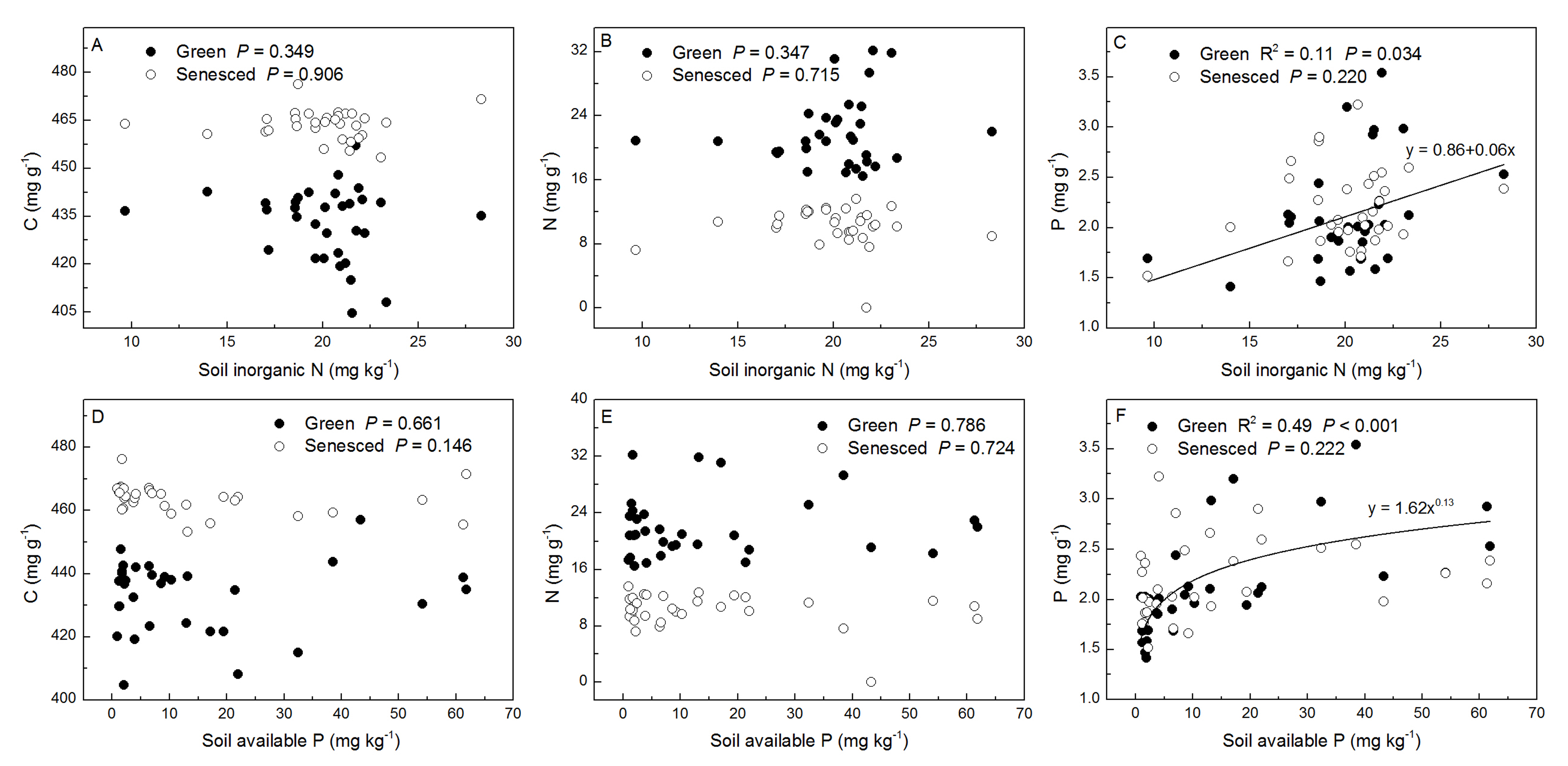


Fig. S4 Relationship between C(A), N(B), P(C) concentration of green and senesced aboveground biomass and soil available nutrient under P additions.


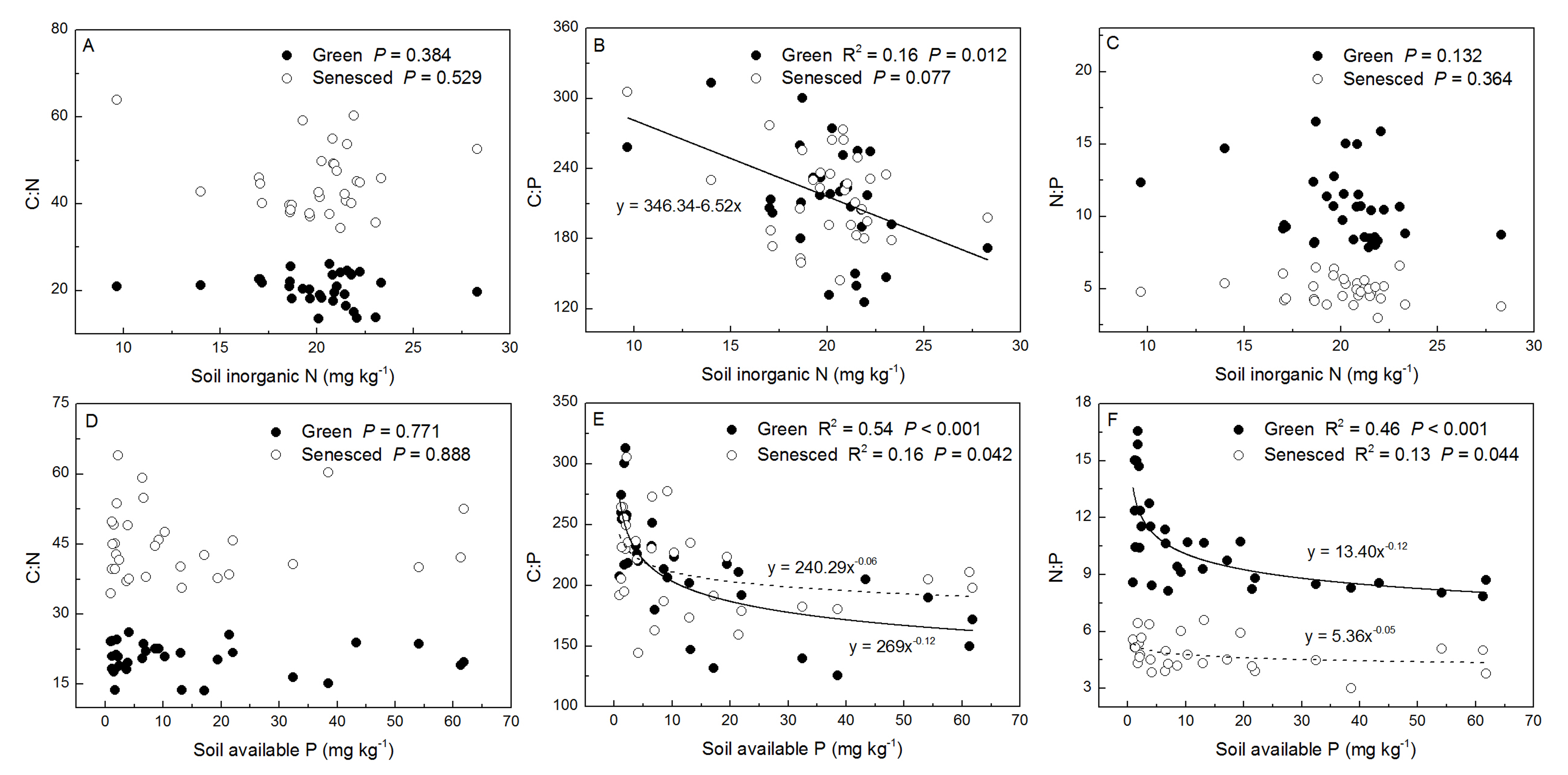


Fig. S5 Relationship between ratios of C, N, P of green and senesced aboveground biomass and soil available nutrient under P additions.
